# A non-pharmacologic approach to address challenging behaviors of Veterans with dementia: description of the tailored activity program-VA randomized trial

**DOI:** 10.1186/1471-2318-13-96

**Published:** 2013-09-23

**Authors:** Laura N Gitlin, William C Mann, W Bruce Vogel, Paul B Arthur

**Affiliations:** 1 Johns Hopkins University, 525 N. Wolfe Street, Baltimore, MD 21205, USA; 2 North Florida/South Georgia Veterans Health System, Center of Innovation on Disability and Rehabilitation Research (CIDRR8), 1601 SW Archer Road (151B), Gainesville, FL 32608, USA; 3 Department of Occupational Therapy, University of Florida, 2107A Health Professions Building, Box 100164, Gainesville, FL 32610-0164, USA; 4 Department of Health Outcomes and Policy, University of Florida, Box 100177, Gainesville, FL 32610-0177, USA

**Keywords:** Alzheimer’s, Dementia, Behaviors, Caregivers, Caregiving, Activity, Veteran, Nonpharmacologic

## Abstract

**Background:**

Behavioral symptoms accompanying dementia are associated with increased health care costs, reduced quality of life and daily functioning, heightened family caregiver burden, and nursing home placement. Standard care typically involves pharmacologic agents, but these are, at best, modestly effective, carry serious risks, including mortality, and do not address behavioral symptoms families consider most distressful and which may prompt nursing home placement. Given dementia’s devastating effects and the absence of an imminent cure, the Veterans Administration has supported the development and testing of new approaches to manage challenging behaviors at home.

**Methods/Design:**

The Tailored Activity Program – Veterans Administration is a Phase III efficacy trial designed to reduce behavioral symptoms in Veterans with dementia living with their caregivers in the community. The study uses a randomized two-group parallel design with 160 diverse Veterans and caregivers. The experimental group receives a transformative patient-centric intervention designed to reduce the burden of behavioral symptoms in Veterans with dementia. An occupational therapist conducts an assessment to identify a Veteran’s preserved capabilities, deficit areas, previous roles, habits, and interests to develop activities tailored to the Veteran. Family caregivers are then trained to incorporate activities into daily care. The attention-control group receives bi-monthly telephone contact where education on topics relevant to dementia is provided to caregivers. Key outcomes include reduced frequency and severity of behavioral symptoms using the 12-item Neuropsychiatric Inventory (primary endpoint), reduced caregiver burden, enhanced skill acquisition, efficacy using activities, and time spent providing care at 4 months; and long-term effects (8 months) on the Veteran’s quality of life and frequency and severity of behavioral symptoms, and caregiver use of activities. The programs’ impact of Veterans Administration cost is also examined. Study precision will be increased through face-to-face research team trainings with procedural manuals and review of audio-taped interviews and intervention sessions.

**Discussion:**

The Tailored Activity Program – Veterans Administration is designed to improve the quality of life of Veterans with dementia and lessen the burden of care on caregivers. Activities are tailored to reflect the Veteran’s preserved capabilities and interests to enhance active engagement, while not taxing areas of cognition that are most impaired.

**Trial registration:**

ClinicalTrials.gov, NCT01357564

## Background

Over 5 million Americans have Alzheimer’s disease or related dementias, a progressive and irreversible neurodegenerative syndrome, with prevalence rates expected to approach 14 million individuals by 2050 in the US [1]. As populations age worldwide, dementia diagnoses are expected to increase dramatically and eventually reach epidemic proportions. Among Veterans 65 and older, rates of dementia are similar to the population at-large, with 7.3% prevalence across all races, except African-Americans, for whom the rate is 50% higher [2]. The prevalence rate of dementia across Veterans Integrated Service Networks (VISNs) ranges from 5.8 to 9.4%, and the disease is associated with substantially higher inpatient and outpatient service utilization relative to other VA patients [2].

Neuropsychiatric symptoms, also referred to as behavioral and psychological symptoms, are a hallmark of dementia, and include agitation, apathy, depression, mood lability, aggressiveness and other behaviors families find challenging, such as wandering, hoarding, disengagement, or rejection or refusal of care [3]. Behavioral symptoms are associated with increased health care costs, reduced quality of life and daily functioning, heightened family caregiver burden, and nursing home placement [4,5]. Standard care typically involves pharmacologic agents, but these are not FDA approved for behavioral management in persons with dementia, are, at best, only modestly effective, carry serious risks including mortality, and do not address the behavioral symptoms that families themselves consider most distressful or prompt nursing home placement [6,7]. Given the disease’s devastating effects and the absence of an imminent cure, medical organizations nationally and internationally, including the VA, support the development and rigorous testing of new approaches to manage behavioral symptoms for persons with dementia living at home.

A promising approach for addressing the behavioral challenges associated with dementia is the use of activity to engage the individual [8]. Preliminary evidence suggests that activities tailored to the interests and capabilities of persons with dementia can reduce behavioral symptoms and improve quality of life [9-11]. However, there is limited research on the effects of using activity at home to reduce behavioral symptoms. Limitations of previous research include a nearly exclusive focus on residents of nursing homes, small sample sizes, and lack of randomized trial designs. As most persons with dementia live at home, testing this approach using randomized trial methodology with diverse populations has the potential for practical real-world clinical application. This report describes a protocol being used in an ongoing randomized trial testing an activity intervention for Veterans living at home with dementia.

## Methods/Design

**Figure 1:**
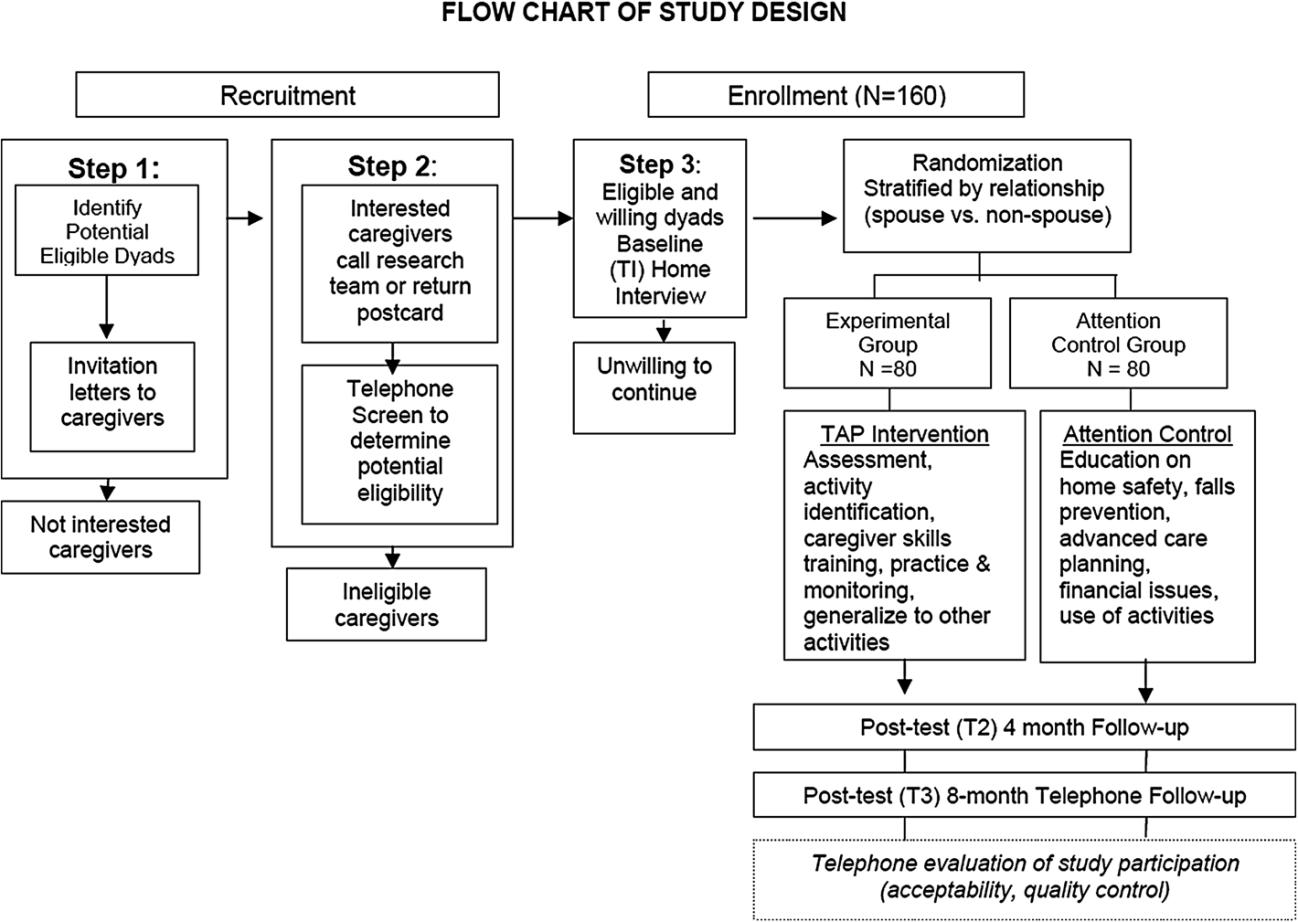
Flowchart of study design.

The TAP-VA study protocol received approval from the Institutional Review Board at the University of Florida and the Research and Development Committee of the North Florida/South Georgia Veterans Health System in Gainesville, Florida (IRB # 454–2011). TAP-VA program staff engaged in readings including a detailed intervention manual and participated in face-to-face 4-day onsite training sessions. All project staff met University and VA standards for research compliance and human subject data collection. A data safety monitoring board was formed, which reviewed and approved all study protocols. General procedures involve a telephone screening of family caregivers to determine eligibility, a home interview to perform baseline data collection, stratified randomization (by caregiver relationship (spouse versus non-spouse) to Veteran), and intervention delivery (Figure 1). All dyads are then retested at 4-months from baseline, main study endpoint, and 8-months from baseline at home to evaluate long-term effects (secondary outcomes).

### Aims and study hypotheses

Our primary study aim concerns the Veteran with dementia and tests the immediate effect of TAP-VA at 4 months on behavioral symptoms. Our hypothesis is that Veterans with dementia who receive TAP-VA will manifest lower total scores on the Neuropsychiatric Inventory (NPI), which assesses frequency and severity of 12 common behavioral symptoms, compared to Veterans assigned to an attention control group.

The study has four secondary aims; one relates to Veteran participants, two relate to caregivers, and one relates to the impact of TAP-VA on health related costs: 1) Test the long-term effects of TAP-VA at 8 months on quality of life and behavioral symptoms of Veterans with dementia. *Hypothesis:* Veterans receiving TAP-VA will manifest higher quality of life and lower total NPI scores over time (baseline to 8 months) in comparison to Veterans in the attention control group; 2) Test the immediate effects of TAP-VA at 4 months and long-term effects at 8 months on caregiver burden, skill acquisition, efficacy using activities, and time spent providing care. *Hypothesis:* Caregivers receiving TAP-VA will report reduced burden, enhanced skills and efficacy using activities, and less time providing care compared to the control group at 4 and 8 months; 3) Examine whether caregivers receiving TAP-VA are using activities at 8 months; and 4) Examine whether TAP-VA results in reduced Veterans Health Administration (VHA) health care use and costs for Veterans with dementia and their caregivers. Results from these secondary aims will provide further evidence of treatment efficacy, identify whether booster sessions are necessary, and inform dissemination efforts and translation of TAP-VA system-wide.

### Setting

If this approach is proven efficacious, our long-term objective is to integrate TAP-VA into standard care practices within the VHA system as the first treatment choice for addressing behavioral symptoms in Veterans with dementia living at home. This would transform the current paradigm of dementia care which relies on pharmacologic management.

The VHA is the largest integrated healthcare system in the nation and provides taxpayer-financed healthcare to honorably discharged Veterans of the United States Armed Forces [12,13]. Parallels are sometimes drawn between the VHA and other nationalized healthcare services, although priority within the VHA is generally granted to Veterans with service-related disabilities, low incomes, and extended terms of service. The VHA maintains a multi-billion dollar electronic health record system that supports communication across its twenty-one Veteran Integrated Service Networks (VISNs) and nearly 300,000 employees [14]. Service connectivity across the VHA allows for numerous sources of referral to TAP-VA to include home care services, rehabilitation services, and physician referral. If TAP-VA is shown to be effective, referral to occupational therapists trained in TAP-VA for home delivery could be offered as a supplemental service within the existing home care structure.

### Study procedures, eligibility and randomization

Our recruitment and enrollment procedures primarily target the caregiver of VA patients in geriatric VA service programs. Recruitment primarily involves mailings to families identified through the VA services. The mailing includes a descriptive IRB approved flyer about the study and a letter from the medical director of the service explaining study procedures and inviting study participation. Caregivers are directed to contact either the research team by telephone or to return a postcard in a self-addressed and stamped envelope indicating an interest in learning more about the study.

Caregivers contacting the research team receive an explanation of study procedures, and if interested in participating in the study, complete a brief telephone screen to determine eligibility. For caregivers eligible and willing to participate, a baseline interview is conducted by a trained interviewer within 2 weeks of determination of eligibility, and prior to randomization to study group. At the time of the interview, the Mini-Mental State Examination (MMSE) is administered to the Veteran. For Veterans with MMSE scores greater than 23, a review of the participant’s medical chart to confirm a current diagnosis of dementia is performed. Following the baseline interview, dyads eligible and willing to participate are randomized (see data analytic considerations) to the TAP-VA treatment group or an attention control group.

Inclusion criteria for Veterans with dementia include: 1) English speaking; 2) diagnosis of dementia; 3) able to participate in at least two activities of daily living (ADLs - bathing, dressing, grooming, toileting, transferring from bed to chair); 4) not currently participating in any other dementia-related intervention; and 5) score of 23 or less on the MMSE or if higher, confirmation of a physician’s diagnosis. Specifically, Veterans with diagnosis codes 290.0, 290.2, 290.3, 331.2 (senile dementias), 290.4 (vascular dementia), 294.8 (dementia not otherwise specified), 331.0 (Alzheimer’s disease), 331.1 (frontotemporal dementia), and 331.82 (Lewy body dementia) are considered to have dementia diagnoses and are thus eligible for study participation.

If the Veteran with dementia is on any of four classes of psychotropic medications (antidepressant, benzodiazepines, antipsychotic, or anti-convulsant) or an anti-dementia medication (memantine or a cholinesterase inhibitor), we require that he/she have been on a stable dose for 60 days (the typical time frame used in clinical trials) prior to enrollment to minimize possible confounding effects of concomitant medications.

Caregivers of Veterans must be: 1) English speaking; 2) self-identify as the primary member of the family caring (hands-on or supervision) for the Veteran; 3) 21 years of age or older (male or female); 4) living with the Veteran; 5) accessible by telephone to schedule interviews, intervention sessions, and follow-up interviews; 6) planning to live in the area for 8 months (to reduce loss to follow-up); 7) willing to learn how to use activities; 8) report one or more behavioral symptoms in the Veteran in the past month; and 9) not currently participating in any other caregiver-related intervention. Finally, we require that caregivers taking a psychotropic medication (e.g., antidepressant, benzodiazepines, antipsychotic, or anti-convulsant) at time of telephone screen be on a stable dose of the medication for 60 days prior to enrollment. A rolling recruitment schedule will continue until 160 Veterans living at home with dementia and whose caregivers report one or more behavioral symptoms are enrolled.

Sample Size is based on: a) one primary outcome (NPI at 4 months); b) treatment effect sizes for outcomes from the TAP pilot study; and c) ability to detect a medium effect size of 0.50. We will use a type I error rate of .05 for the single primary hypothesis. We seek to detect a moderate effect size (a 0.5 standard deviation difference). A smaller effect size brings us at or near levels where the study could have statistical but not clinical significance [i.e., number needed to treat (NNT) of 4.5]. In order for an intensive, targeted intervention to be worth implementing, it must yield more than trivial effects.

Since our planned analyses are primarily analyses of covariance with baseline as the covariate, we calculate the power of a two-sample t-test on change scores from baseline to 4 months. Because we will be using analyses of covariance to adjust for baseline, we expect to have greater precision and power than indicated here. To attain 80% power for a two-sided alternative hypothesis using a t-test comparing the two treatment groups with respect to 4-month values will require 64 dyads per group. However, we plan to recruit an additional 16 per group or 32 dyads for a total of 160 (80 per group). Thus, to obtain the necessary sample size of 128, 160 will need to be enrolled.

Veterans with dementia diagnostic codes (listed in inclusion criteria) are recruited from the North Florida/ South Georgia Veterans Health System Geriatric Research, Education & Clinical Center and Geriatrics and Extended Care Service outpatient services, including the Home Based Patient Care and Homemaker Home Health Aide program. Our recruitment efforts will also include independent senior living facilities, adult day services, and support groups.

All eligible dyads receive a baseline home interview (Table 1) and then are randomized to either the experimental (TAP-VA) or attention-control groups. Randomization is stratified by the caregiver’s relationship to the Veteran (spouse vs. non-spouse) to ensure that the two intervention groups are balanced with respect to this factor. Type of relationship has previously been associated with treatment processes, outcomes, and study attrition [15]. For randomization within each stratum, the method of random permuted blocks is used to control for possible changes over time in the subject mix. The blocking number was developed by the project statistician, and is not disclosed to the investigators or other members of the project team. The project statistician provides the necessary materials and randomization list to a research staff member who is not involved in study oversight, intervention, or interview. This individual prepares consecutively numbered randomization envelopes which contains the group allocation information on a piece of paper folded over multiple times to obscure the information, and provides the envelopes to the project coordinator. The project coordinator then randomizes a subject by opening the next available envelope for the appropriate stratum (spouse/non-spouse). The randomization procedure occurs after the baseline assessment. Families who do not wish to continue in the study after the interview will not be randomized and not count towards accrual goals.

**Table 1:** Assessment measures

**Measure**	**Purpose**	**When assessed**
Investigator-Developed Semi-Structured Interview	Collects demographic information, CG health and behavior, confidence in meaningful activity, vigilance, mastery and control, leisure frequency and amount, CG and Veteran medication use.	Baseline (T1) Interview,
		4-Month (T2) Interview,
		8 Month (T3) Interview
Mini Mental State Exam (MMSE)	Screening to detect cognitive impairment. Also used to verify inclusion criteria.	Baseline (T1) Interview
Caregivers Assessment of Functional Dependence and Upset (CAFU)	Measures dependence in Veteran with dementia and caregiver reaction.	Baseline (T1) Interview,
		4-Month (T2) Interview,
		8 Month (T3) Interview
Neuropsychiatric Behavior Inventory (NPI-C)	Measures behavioral disturbances in Veteran with dementia.	Baseline (T1) Interview,
		4-Month (T2) Interview,
		8 Month (T3) Interview
Quality of Life AD (QOL-AD)	Detects a rating of the Veteran's quality of life.	Baseline (T1) Interview,
		4-Month (T2) Interview,
		8 Month (T3) Interview
The Center for Epidemiologic Studies Depression Scale (CES-D)	Measures the depressive symptomology of the caregiver.	Baseline (T1) Interview,
		4-Month (T2) Interview,
		8 Month (T3) Interview
Zarit Short Form Burden Scale	Measures caregiver burden in relation to time, developmental comparison with peers, physical health, social relationships, and emotional health.	Baseline (T1) Interview,
		4-Month (T2) Interview,
		8 Month (T3) Interview
Task Management Strategies Index (TMSI)	Identifies actions taken by caregivers to simplify everyday self-care tasks for individuals with dementia.	Baseline (T1) Interview,
		4-Month (T2) Interview,
		8 Month (T3) Interview
Dementia Management Strategies Scale (DMSS) Short Version	Identifies the frequencies with which caregivers use a variety of management strategies to deal with dementia-related behavior problems.	Baseline (T1) Interview,
		4-Month (T2) Interview,
		8 Month (T3) Interview
Allen Diagnostic Module ( 2^nd^ Edition)	Provides opportunities to observe global cognitive processing capacities, learning potential, and performance abilities to detect unrecognized or suspected problems of functional cognition.	OT Intervention, Period 1

Interviewers are masked to group allocation. Interviewers and interventionists do not share office space and staff meetings do not involve discussion of study participant allocation. Additionally, caregivers are asked not to discuss their group assignment with interviewers. Following each follow-up interview, the interviewer is asked to record which study group they believe the dyad was assigned, and the reason for their guess. This provides a way to monitor assignment disclosures and track any off protocol occurrences with regard to unmasking.

### Recruitment progress

Recruitment procedures have been implemented and will continue for a total of 33 months, with approximately 5 dyads enrolled per month. To date, we have enrolled 41 participants from 362 initial mailings sent starting April, 2012, and again to non-respondents in August, 2012. Of the first two mailings, 64 (17.6%) responses were received via telephone or postcard, of which 41 (64%) were eligible, willing to participate, and enrolled. This recruitment rate mirrors our initial goal of 5 dyads per month. An additional 185 mailings have been sent in June and July of 2013 to newly identified Veterans. Eleven letters have also been sent to local rehabilitation clinics, VA clinics, and adult care facilities for study outreach and to aid recruitment.

Reasons for ineligibility have included dependent feeding (bringing food to mouth), disinterest after telephone screen, imminent nursing home or hospice placement, move outside of study radius, and death of Veteran with dementia. Attrition between baseline and randomization is currently 2 (4.8%). Attrition after randomization is currently 11(26.8%) due to: Veteran death [4], Veteran illness [4], and caregiver-initiated withdrawal [3]. This is slightly above our expected overall attrition rate of 20%.

### TAP-VA intervention

The intervention is designed to draw on spared or residual abilities of Veterans with dementia and provide an environment supportive of these abilities. Occupational therapists (OTs) conduct an assessment of the person’s home environment, preserved capabilities, daily routines and interests and the caregiver’s readiness and ability to use activities. This assessment uses a combination of caregiver and Veteran self-report, direct observations and performance-based tests. Based on the assessment, activities are developed that reflect the Veteran’s previous or current interests and are then modified to match the person’s preserved capabilities without taxing the most impaired areas of cognition (e.g., memory, new learning). In designing activities, the occupational therapist interventionists simplify an activity and structure it to focus on single rather than multiple or complex tasks, thereby minimizing potential for the Veteran to make errors.

The activity environment is constructed to provide auditory or tactile cues to facilitate recall and guide initiation and sequencing. By grading activities to match the Veteran’s capabilities, the occupational therapist interventionists minimize demands that may heighten stress (e.g., high functioning individuals will be introduced to more goal-directed and multi-step activities, whereas lower functioning individuals will be introduced to activities that are based on repetitive motion (e.g., washing windows, folding towels) and integrate multi-sensory stimulation (e.g., soft music, objects pleasant to touch). Whereas other activity interventions may emphasize new learning, our approach does not, although it may entail some procedural learning if appropriate.

TAP-VA provides caregivers with the requisite knowledge and skills to use activities. Caregivers are instructed in specific skills such as ways to simplify activities, the environment and their communication, and how to help the Veteran initiate and follow a sequence. The overall goal is to provide predictability, familiarity, and structure in the daily life of the Veteran and establish a level of environmental stimulation appropriate to that person’s abilities. Three clinical elements are used to enhance caregiver receipt and enactment of intervention strategies: 1) the interventionist engages caregivers (versus being prescriptive) in learning how to use activities by modeling, simulating, role play and direct demonstration with the Veteran, 2) the caregiver is provided repeated opportunities for practice and modification of techniques, and 3) the interventionist uses validation and actively demonstrates techniques within the context of their use.

To enhance effective use of activity, caregivers are provided concrete knowledge as to what the Veteran is capable of doing (e.g., can grasp object such as a plate) and his/her specific limitations (e.g., needs verbal cueing to place dish in cabinet). With this knowledge, interventionists can help caregivers restructure their expectations, behavioral goals, and reactions to the Veteran. During treatment sessions, caregivers are taught to: 1) recognize capabilities and deficits, 2) translate capabilities into objective activity goals, and 3) generate specific steps to set up activities and help caregivers initiate/sequence.

### Attention control group

The attention control group serves three purposes: 1) creates clinical equipoise, ensuring that ethical treatment is provided to all study participants; 2) controls for the one-on-one attention to caregivers in the Tailored Activity treatment group to rule out potential effects of professional contact; and 3) serves as a retention tool to keep control group caregivers meaningfully connected to the trial. Caregivers in this group receive bi-weekly telephone contact (up to 8 contacts) by a trained healthcare professional. In each session, caregivers are provided important information about dementia and strategies for managing the disease at home (Table 2). Each telephone contact is approximately 30 minutes in length and begins with a brief overview of the specific purpose of the session, followed by a description of the key facts about the session topic, and concludes with a question and answer period. Table 2 outlines the specific domain and session content that is covered. The attention control group intervention is delivered by a member of the research team who is knowledgeable about dementia and has had prior experience working with family caregivers.

**Table 2:** Attention-control group

**Domain**	**Brief description of content**
Education About dementia	Overview of key facts about dementia as a degenerative disease that currently has no cure.
General Safety Issues	Information about safety issues inside and outside the home, the types of safety hazards to be aware of, and how to make the environment safer for the Veteran and caregiver.
Home Safety Room-by-Room	Information about preventing accidents by doing a room safety check in the home and tips for making each room safer for the Veteran and caregiver.
Being a Medical Advocate for your Veteran	Suggestions for the caregiver about working with healthcare providers and other members of the health care team to ensure the Veteran receives the best care possible.
Taking Care of Yourself as a Caregiver	Tips for the caregiver about taking care of their own social, physical, recreational, and other needs so that they may continue to provide quality care for their Veteran.
Behavior Problems	Education about behavior problem areas and suggestions for preventing or mediating unwanted or harmful behaviors associated with dementia.
Activities for the Veteran and Caregiver	Suggestions for engaging the Veteran and caregiver in activities and information about that benefits from participation in activities designed specifically for them.
Summary	Brief summary of the previous 7 session topics and an opportunity for caregiver feedback about educational sessions.

### Analytic plan

Descriptive analyses and univariate comparisons of the experimental and control group conditions using Chi-square and Wilcoxon rank-sum tests will be performed as appropriate. If any imbalance (large or statistically significant difference) is found on important prognostic factors, those factors will be forced into all ANCOVA’s. Chi-square and Wilcoxon rank-sum tests are performed as appropriate to identify differences between experimental and control group at baseline. In addition to serving as a final data quality check, these analyses are used to characterize the study population, assess the success of randomization in balancing the two groups, and determine the impact of any dropouts on that balance with respect to potentially important prognostic factors (e.g., age, education level of Veteran, MMSE score).

Experiences in earlier trial phases suggest that attrition is an external, rather than an internal validity problem. That is, while those who drop out differ from those who remain, the control and intervention groups remain comparable. This is an important consideration for analysis, so the internal validity comparison is part of these initial analyses. Experimental and control group subjects who remain at T2 are compared on their baseline characteristics to determine whether the groups remain comparable (internal validity). This comparison also occurs for dyads remaining at T3. If any imbalance (large or statistically significant difference) is found on important prognostic factors, highlighted factors will be forced into all ANCOVA’s.

Covariates such as comorbidities, disease stage, psychotropic medication use, caregiver relationship, and gender are also considered. Although it may be of interest to evaluate treatment effect on long-term/permanent nursing home placement, significant sample size and recruitment might not be possible. All analyses utilize current versions of SPSS, SAS, and Statistica.

The primary endpoint is the NPI index (total summary score reflecting frequency by severity) at 4-months. The focus of the primary analysis is to examine the effect of the intervention based on "intention to treat." Data from all dyads, randomized to the experimental group, will be part of the primary analyses regardless of actual number of completed intervention sessions. This approach tests the effect of the intervention without taking into account the extent to which dyads actually receive intervention and effectively penalizes the intervention if dyads are not willing to receive it or if dyads receive different doses or exposure to treatment. The adjusted mean differences in treatment effects on the outcome measure at 4 months are calculated using analysis of covariance. Covariates include the baseline value of the outcome measure, the stratification variable (spouse vs. non-spouse) and other background characteristics if large or statistically significant differences between experimental and control group dyads are recognized.

For the ANCOVA analyses, a test of the normality assumption for the dependent measure is performed by examining distribution of residuals. If the residual distribution is skewed, a transformation of the data will be used. A similar approach will be used for secondary aims. Examination of whether TAP-VA has a long-term treatment effect at 8 months (T1-T3) on total NPI index is performed by using ANCOVA analysis controlling for baseline, stratifying and other potential covariates (e.g., comorbidities). The other secondary endpoints address caregiver burden (Short Zarit Burden scale, upset with behaviors, and time spent in care (2 vigilance items), and skill acquisition (Task Management index)). The same analytic procedure as described for Aim 1 is followed for T2 and T3 data analysis.

### Cost-effectiveness analysis

A cost-effectiveness analysis of the TAP-VA intervention treatment group compared to the attention control group will be performed using an innovative approach for constructing incremental cost-effectiveness ratios [16]. Rather than using Quality-Adjusted Life Years (QALYs) as the measure of effectiveness, cost-effectiveness is measured as the cost of achieving one additional unit of benefit as defined by caregiver hours per day “doing things” and hours per day “being on duty.” These measures have advantages over QALYs in this application, including: 1) their focus on a proximate caregiver outcome of the TAP-VA intervention and 2) the ability to compare this cost per unit time measure to both caregiver opportunity costs and willingness-to-pay estimates from previous studies [17], thereby avoiding issues related to the possible lack of sensitivity of QALY measures to the intervention and debates over appropriate QALY valuation thresholds. These two time measures are derived from the 4-item Caregiver Vigilance Scale used in the Resources for Enhancing Alzheimer’s Caregivers Health (REACH I) study [18].

Total costs of care for both groups will be compared using a combination of existing VA patient-specific cost data, direct cost measurement, and patient/caregiver cost diaries. VA patient-specific costs originate from the VHA’s Decision Support System (DSS) National Data Extracts (NDEs) [19]. DSS uses a traditional cost accounting system where specific costs are assigned to specific cost centers, and costs from indirect cost centers (commonly thought of as “overhead”) are allocated to direct cost centers (those which produce patient care) using standard step down allocation methods. For those elements of the TAP-VA intervention not adequately captured in the DSS NDEs, direct cost measurement will be used [20,21]. In addition, cost diaries [22] will be used to capture the indirect costs associated with care-related travel, work-loss days, and out-of-pocket expenses incurred by the Veteran and caregiver.

Validation of the DSS NDE cost data will be performed by merging DSS with the VA's Health Economics Resource Center (HERC) Average Cost Dataset, which provides encounter-level regression- and relative value unit-based VA cost estimates using Medicare cost structures applied to VA patient and facility characteristics. This is in accordance with HERC guidance, which recommends using the HERC Average Cost data as a validation check for the DSS cost data. In addition, costs for patients who are eligible for and receive Medicare services will be obtained using actual Medicare payments from CMS Medicare data provided through the VA Information Resource Center (VIReC). While Medicare payments include a nominal profit margin, such margins are specifically sized to approximate a normal return on providers’ invested capital, and hence can be taken as approximate measures of the total (variable plus fixed) costs of care. Nevertheless, because Medicare payments are qualitatively different from VHA DSS costs, these Medicare dollars will be tallied separately from and compared to VHA costs of care. If statistically significant differences are found, the sensitivity of cost results to the use of Medicare payments vs. VHA costs are explored further.

As it is difficult to distinguish costs specifically influenced by the intervention using observational data, cost is assessed over a 12 month period by summing all inpatient and outpatient costs for each patient in the sample. By so doing, external or “spillover” cost effects of the intervention will be captured, thereby providing a more accurate and comprehensive measure of the impact of the intervention. Cost models will follow customary practice and use the natural logarithm of patient-specific costs as the dependent variable to account for the inherent skewness of cost data. Duan’s smearing retransformation estimator corrected for heteroskedasticity will be used in calculating cost predictions from the models. Because the sample consists of chronically-ill patients, it is not anticipated that any subject will have zero costs in the data. Consequently, there is not an expected need to estimate a two- or four-part model to account for any large probability mass at zero costs. However, if a significant number of patients with zero values in the time-specific cost variables exists, a two- or four-part model will be employed as necessary. The cost hypothesis will be tested using the F-test in the cost regressions for the overall rehabilitation settings construct, supplemented by the t-tests for each setting. If the incremental cost-effectiveness ratio measures prove sensitive to denominators close to zero in absolute value, the treatment and control arms are compared using the net monetary benefit approach in order to avoid the discrepancy [23].

## Discussion

TAP-VA is testing the effectiveness of an individualized Veteran-centric intervention to reduce behavioral symptoms and improve quality of life. Secondarily, we will determine the potential reduction in time caregivers spend in care, and increase in caregiver skills as they relate to incorporating activities into daily routines. We also seek to evaluate potential cost savings in terms of health care utilization rates of both Veterans and family caregivers using a cost diary. Current recruitment rates imply sufficient design and planning and suggest moderate population interest in the program. Attrition rates to date are modest and as expected for the population due to their complex medical diagnoses and care situations associated with dementia and aging. The employed design is an advancement over previous activity studies in that it focuses on persons at home, tests an approach that families can use themselves in daily care, adheres to randomized trial methodology, and includes a prospective cost analysis that will enhance its eventual translation and implementation potential.

If TAP-VA is efficacious and cost-effective, it will represent a promising approach for addressing behavioral symptoms associated with dementia and caregiver burden, warranting replication and implementation within the Veterans Administration, as well as other healthcare systems. In conclusion, TAP-VA is underway with a Veteran population, though the originating intervention components and principles have high transferability to other dementia populations and settings.

## Abbreviations

TAP-VA: Tailored Activity Program Veterans Administration; VA: Veterans Administration; VISNs: Veterans Integrated Service Networks; VHA: Veterans Health Administration; NPS: Neuropsychiatric Symptoms; VISN: Veterans Integrated Service Network; NPI: Neuropsychiatric Inventory; MMSE: Mini Mental State Examination; ADL: Activities of Daily Living; NNT: Number Needed to Treat; QALYs: Quality-Adjusted Life Years; REACH: Resources for Enhancing Alzheimer’s Caregivers Health; DSS: Decision Support System; NDEs: National Data Extracts; HERC: VA Health Economics Resource Center; VIReC: VA Information Resource Center.

## Competing interests

The authors declare that they have no competing interests.

## Authors’ contributions

LNG, investigator, developed study concept and primary design, developed research questions, developed intervention and model, assumed primary conceptual development and writing responsibility of manuscript. WCM, principal investigator, adapted study concept to Veterans Administration guidelines, oversaw scientific integrity and interpretation of data, and provided critical review of the manuscript. WBV, research health scientist, assisted in cost analyses, interpretation of cost data and wrote cost description and provided a critical review and editing of manuscript. PBA, research assistant, responsible for participant screening, collection of baseline and reassessment data, coordinating manuscript development and review, and assisting in the creation of tables and figures. All authors read and approved the final manuscript.

## Authors’ information

LNG, Director, Center for Innovative Care in Aging, Johns Hopkins University.

WCM, Director, Center of Innovation on Disability and Rehabilitation Research (CIDRR8), North Florida/South Georgia Veterans Affairs; Distinguished Professor and Chair, Department of Occupational Therapy, University of Florida.

WBV, Research Health Scientist, Center of Innovation on Disability and Rehabilitation Research (CIDRR8), North Florida/South Georgia Veterans Health System; Associate Professor, Department of Health Outcomes and Policy, University of Florida.

PBA, Research Assistant, Center of Innovation on Disability and Rehabilitation Research (CIDRR8), North Florida/South Georgia Veterans Health System; Predoctoral Research Fellow, Department of Rehabilitation Science, University of Florida.

## Pre-publication history

The pre-publication history for this paper can be accessed here:

http://www.biomedcentral.com/1471-2318/13/96/prepub
